# Novel Expansible Aortic Annuloplasty Ring Exhibits Similar Characteristics as the Dacron Ring—an In Vitro Evaluation

**DOI:** 10.1007/s12265-023-10393-7

**Published:** 2023-06-01

**Authors:** Mariam Abdi Noor, Leila Louise Benhassen, Alexander Emil Kaspersen, Marc Gjern Weiss, John Michael Hasenkam, Peter Johansen

**Affiliations:** 1 Department of Clinical Medicine, Aarhus University, Aarhus, Denmark; 2 Department of Cardiothoracic and Vascular Surgery, Aarhus University Hospital, Aarhus, Denmark; 3 Department of Electrical and Computer Engineering, Aarhus University, Aarhus, Denmark; 4 Department of Surgery, University of the Witwatersrand, Johannesburg, South Africa

**Keywords:** Aortic valve repair, Annuloplasty, Hemodynamics, Cardiovascular interventions

## Abstract

An increasing body of research indicates that annular stability plays a key role for a successful aortic valve repair. The aim of this study was to evaluate and compare a novel open aortic annuloplasty ring (the A-ring) with the Dacron ring. Both rings were compared with native aortic roots in vitro. Eighteen aortic roots were included in the study and randomized into three groups: the native, Dacron, and A-ring group. The roots were evaluated in an in vitro physiologic pulsatile model simulating the left side of the heart. Aortic annulus diameters were significantly reduced both in the Dacron ring group (*p* = 0.003) and the A-ring group (*p* = 0.020) when compared with the native group. Both the Dacron ring and A-ring effectively downsized the aortic annulus diameter. The A-ring also displayed an ability to maintain aortic root distensibility during the cardiac cycle equally to the Dacron ring.

**Graphical abstract:**

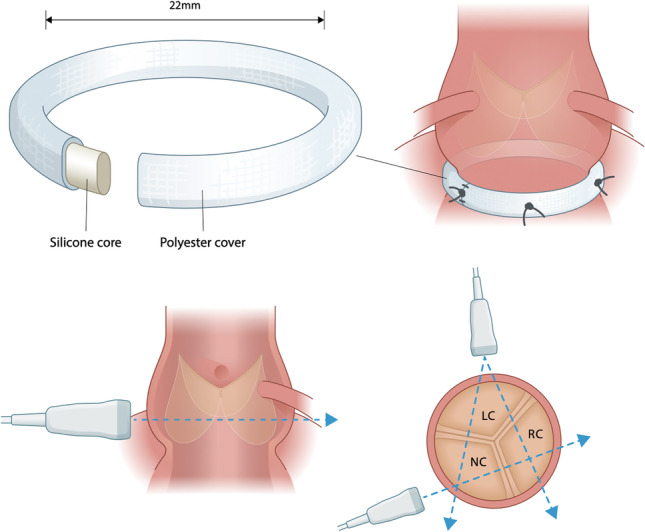

**Supplementary Information:**

The online version contains supplementary material available at 10.1007/s12265-023-10393-7.

## Introduction

For patients suffering from aortic regurgitation, with or without aortic root dilatation, the traditional treatment of choice has been aortic valve replacement. However, within the last two decades, aortic valve repair has emerged as a preferable treatment without the complications associated with prosthetic valve replacement. This entails the risk of endocarditis, lifelong anticoagulant therapy in mechanical valve recipients, or structural bioprosthetic valve degeneration potentially requiring reoperation [1].

One of the main risk factors for failure after aortic valve repair is an untreated dilated aortic annulus greater than 25–28 mm [2–4]. To achieve an optimal valve repair, increasing data suggests that the aortic annulus should be stabilized to prevent further root dilatation and recurrent aortic regurgitation [5]. At short and medium follow-up, clinical trials have demonstrated that an annuloplasty ring may decrease aortic annulus diameter, increase coaptation height [6, 7], and improve freedom from recurrent aortic regurgitation [8].

Currently, there is no gold standard for an aortic annuloplasty in terms of material, shape, or position. However, one of the clinically most used annuloplasty rings is the Dacron ring, which is made from a Dacron tube graft [6]. Due to the material characteristics of the Dacron ring, concerns have emerged regarding the potential for the Dacron material to become stiff over time and loose its expansibility throughout the cardiac cycle. Basmadjan et al. showed a decrease in systolic expansion of the aortic annulus after implantation of a Dacron annuloplasty ring from 17% at discharge to 9% after 2 years [8]. Although the findings were statistically and clinically non-significant, further long-term follow-up studies are warranted.

Various surgical techniques have been used to repair a dilated aortic root [9–13], all aiming at finding the ideal method to stabilize the aortic root in an effective and durable manner, as well as reestablishing biomechanical properties similar to the native aortic root [13, 14]. It has previously been shown that a flexible aortic root is essential for the natural physiological movement of the aortic valve leaflets and the aortic annulus [15]. Therefore, to optimize the dynamic function of the aortic annulus, a flexible material should be considered for the annuloplasty ring to allow the natural movements of the native aortic root. Various techniques and materials have been proposed for an annuloplasty ring, but their clinical use remains limited. Lansac et al. developed and reported promising results on a flexible extra-aortic ring. However, Lansac’s annuloplasty procedure comprised a closed ring and can therefore only be used in patients having valve-sparing aortic root procedures and not for isolated aortic valve repair where the coronary arteries are not detached and reimplanted.

This current study presents a new type of flexible annuloplasty ring termed the A-ring. The A-ring was designed to provide a stable and comprehensive annuloplasty while maintaining aortic root distensibility [16]. The A-ring is open and designed for the purpose of reducing the diameter of a dilated aortic annulus during diastole while preserving systolic root distensibility. In the present study, the A-ring will be compared with the already used Dacron ring. We hypothesized that the A-ring would downsize the aortic annulus while increasing the coaptation length, reducing the tenting area, and maintaining the overall aortic root dynamics, equally to the Dacron ring. Thus, the aim of the study was to characterize the overall function and dynamic properties of the aortic root after implantation of a Dacron ring and the A-ring and compare both interventions with a native aortic root control group.

## Materials and Methods

### Study Material and Surgical Preparation

Eighteen fresh porcine hearts from 80 kg pigs were collected from a slaughterhouse and stored in a cooler at 3 °C. The aortic roots were randomized using an online software (List Randomizer, Random.org) into three groups: the Dacron ring group, the A-ring group, and the native aortic root group (control group).

**Fig. 1:**
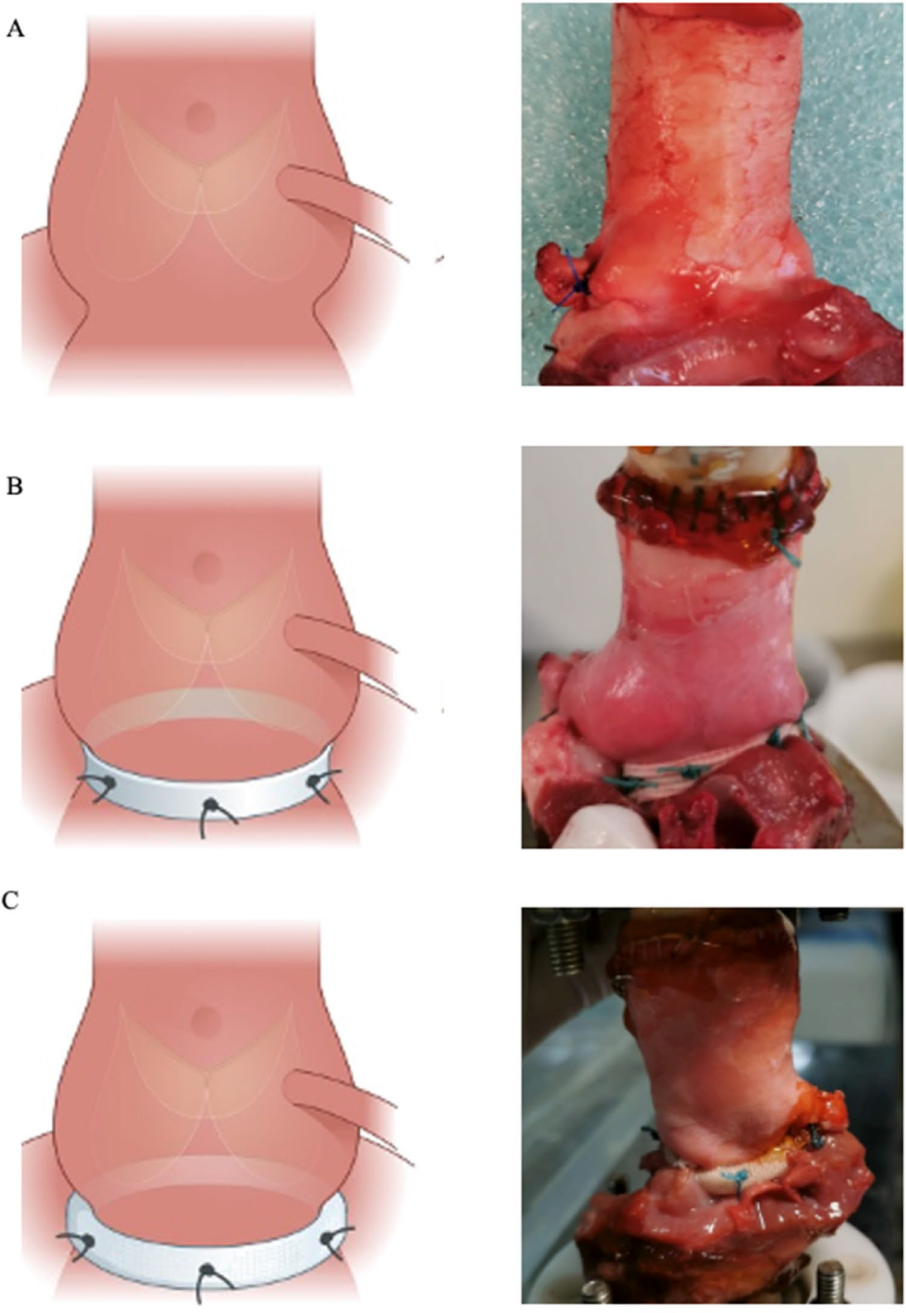
Schematic illustration and photography of the annuloplasty procedures. **A** Native aortic root. **B** Dacron ring annuloplasty. **C** A-ring annuloplasty

Preparation and in vitro evaluation of aortic roots from porcine hearts were performed as previously described [17]. The aorta was transected 2 cm downstream of the sinotubular junction and the left ventricle was cut off 3 cm upstream of the aortic annulus. The aortic roots were inspected, and only aortic roots with three normal aortic cusps were included in the study. The intraluminal diameter of the aortic annulus was measured using Hegar dilators, and aortic roots with an internal diameter of 23 mm were included. The annular base of the aortic root was dissected free, and the coronary arteries were ligated proximally. A uniform and standardized approach for placing the sutures was used for the annuloplasty procedures as illustrated in Fig. 1.

For the Dacron ring procedure (Fig. 1B), a circular band with a height of 4 mm was cut from a straight Dacron tube graft having a diameter of 22 mm (Gelweave, Vascutek Ltd., Renfrewshire, UK). The diameter of the Dacron ring was derived from the sizing criterion proposed by Lansac et al. [18]. The A-ring consists of a silicone core enclosed by a polyester textile with a height of 4 mm. The diameter of the A-ring was based on the same sizing criterion as the Dacron ring, thus also having an internal diameter of 22 mm. Both interventions were tied down and fastened in the same manner. This approach was also described by Lansac et al. [19]. Six anchoring “U” stiches were passed inside-out of the aortic wall and through the ring at the subvalvular plane using 2–0 Ethibond sutures without felt (Ethicon, Inc., Somerville, NJ, USA). A final suture was used to close the rings since we used water as a test fluid. A surgical adhesive (BioGlue, CryoLife Inc., GA, USA) was used between the stiches and the graft to ensure a tight and competent sealing. Lastly, a 3-cm-long Dacron tube was sutured to the ascending aorta and left ventricular outflow tract to provide best mounting of the aortic root into the in vitro set-up.

### *In Vitro* Set-up

**Fig. 2:**
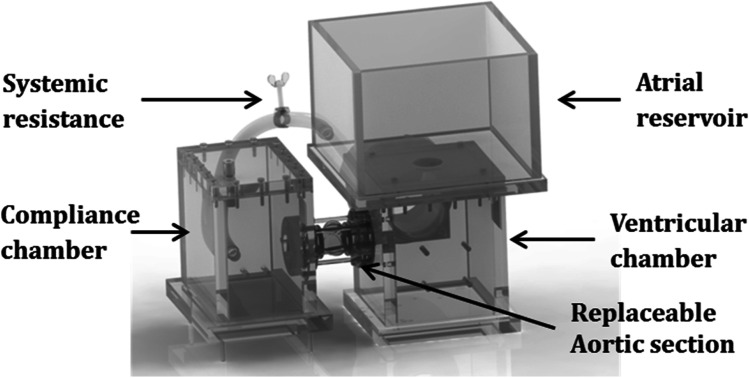
An illustration of chambers and peripheral units in the in vitro pulsatile model

A pulsatile left heart flow loop was used for the experiments (Fig. 2). The in vitro model comprised an atrial chamber and a ventricular chamber connected by a mechanical mitral valve. The ventricular chamber was coupled to a digitally controlled piston pump (SuperPump AR Series, ViVitro Labs, Victoria, Canada), delivering a pulsatile flow to simulate left ventricular ejection into the aortic root. The aortic root was mounted in an exchangeable aortic section in the model and a compliance chamber was mimicking arterial elasticity (Windkessel). The systemic vascular resistance was fine-tuned through an adjustable clamp to adjust for the target systemic pressures.

Aortic flow and peripheral venous flow were measured using ultrasonic transit time flowmeters (PXL11, PXL25, TS410, Transonic Systems Inc., NY, USA). Pressures in the left ventricular chamber and the arterial compliance chamber were measured using Mikro-Tip pressure catheters (SPR-350S, Millar Instruments, TX, USA) and amplified using a 2-channel pressure control unit (PCU-2000, Millar Instruments). Systolic and diastolic pressures were targeted to 120/75 ± 5 mmHg, and the flow was targeted to 5 ± 0.5 L/min throughout all experimental runs.

The pressure and flow signals were digitized at a sample rate of 1 kHz (cDAQ model 9172, NI-9237, NI-9215, National Instruments, Austin, TX, USA). The recorded data were collected using custom made software (LabVIEW 11.0, National Instruments), and continuously sampled during the acquisition for 20 s. Throughout the sampling and recording time, the pressure and flow curves were at the same time graphically represented for online monitoring.

Images of the aortic valve were obtained using a high-speed camera operating at 125 frames per second with a resolution of 1024 × 1024 (FASTCAM SA3, Photron Inc., CA, USA). The images were displayed and recorded using the Photron FASTCAM Viewer software. The images were only used to qualitatively assess the installment of the aortic root and to visualize the valve leaflet movements. The total data collection time for each aortic root was 30 min.

### Echography

**Fig. 3:**
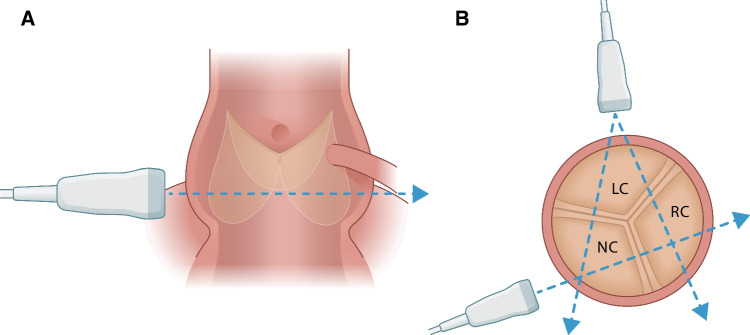
Schematic illustration of the aortic root with all echographic projections. **A** The aortic root in the short-axis view. **B** The aortic root in the long-axis view. NC, non-coronary; RC, right coronary; LC, left coronary

Echographic evaluation of annular and leaflet dynamics was achieved using a two-dimensional linear probe (GE 9 l-RS Probe, GE Vingmed Ultrasound AS, Horten, Norway). One short-axis view was acquired at the sinus plane and three long-axis views across the adjacent leaflets (left/right coronary, right/non-coronary, and left/non-coronary interleaflet triangles) visualizing the entire aortic root (Fig. 3). A mean value was calculated from the three long-axis views for each group and used for comparison between groups.

Definition of echographic systole and diastole was derived from the maximum and minimum annular diameters, respectively. The obtained echographic parameters of the annulus were defined from the internal diameter at the level of the nadir of the three leaflets (systole and diastole); mid-sinus internal diameter (systole and diastole); coaptation length (length of direct leaflet contact); tenting area (area between the aortic annulus and the lower border of leaflet coaptation); geometric orifice area (planimetric opening area formed by the free edges of the leaflets in the systole) [20]; and planimetric cross-sectional sinus area (entire area at the level of the sinuses) [17]. The echographic measurements were performed visually. The likelihood of operator-induced bias was overcome by blinded analysis, and intra- and interobserver variability were less than 10%.

### Study Design

All aortic valves were inspected for competency through echography and videos of the aortic root and valve were monitored using a high-speed camera. The first data collection comprised hydrodynamic measurements (pressures and flow) to assess transvalvular pressure gradient and effective orifice area. The second data collection was performed using two-dimensional echography for obtaining the echographic measurements in long- and short-axis views.

### Data Analysis

Data analyses of the pressure and flow signals were handled using custom-made software (LabVIEW 11.0, National Instruments, Austin, TX, USA). Hydrodynamic analyses were based on ten consecutive cardiac cycles for each aortic root. The systolic and diastolic pressures were determined from maximum and minimum of the aortic pressure curves, while the transvalvular pressure gradient (Δ*P*) was derived from the time of peak aortic flow to achieve an instantaneous transvalvular gradient [20]. The effective orifice area (EOA) was calculated using Gorlin’s Eq. [21] from the minimal cross-sectional area of the flow jet:

$$\mathrm{EOA}=\frac{Q}{51.6 \sqrt{\Delta P}}$$

where *Q* was the mean aortic flow measures by the transit time flow meter.

Echographic data was analyzed using OsiriX MD v12.0 (Pixmeo Sarl, Bernex, Switzerland). The echographic analysis was performed using inner-edge to inner-edge measurements [22]. The distensibility was calculated based on the values derived from the difference between the systolic and diastolic diameters at the respective levels [17].

### Statistical Analysis

All data are presented as mean ± standard deviation following assessment of normal distribution. The assumption of normality was assessed by inspection of normal quantile plots and tested with the Kolmogorov–Smirnov test. The pressure and flow data obtained from the hydrodynamic measurements were analyzed using general mixed-effects models for repeated measures with group and cardiac cycle as fixed effects, and animal as a random effect. The echographic data was analyzed using one-way ANOVA with Bonferroni corrected post hoc tests, where the assumption of homogeneity of variance was checked using Levene’s test. All tests were two-tailed and interpreted at a statistical significance level of 0.05. The statistical analyses were performed using SAS® Enterprise Guide® software, version 7.1 (SAS Institute Inc., Cary, NC, USA).

## Results

### Hydrodynamics

All aortic roots were competent at all time points. The hydrodynamic measurements are presented in Table 1. Flow and systolic and diastolic pressures were all kept within the predefined target ranges. The transvalvular pressure gradients were significantly higher in the Dacron ring group (*p* < 0.001) and in the A-ring group (*p* < 0.001) compared with the native group. The effective orifice area significantly decreased in the Dacron ring group (*p* < 0.001) and in the A-ring group (*p* < 0.001) compared with the native group. There was no significant difference in transvalvular pressure gradient or effective orifice area between the Dacron ring group and the A-ring group.

**Table 1:** Hydrodynamics obtained from all 18 porcine hearts

	Native (*n* = 6)	Dacron (*n* = 6)	A-ring (*n* = 6)
	Mean ± SD	Mean ± SD	Mean ± SD
*Q*_mean_ (L/min)	5.2 ± 0.2	5.3 ± 0.3	5.5 ± 0.2
*P*_systolic_ (mmHg)	120 ± 3	120 ± 1	119 ± 1
*P*_diastolic_ (mmHg)	80 ± 11	76 ± 6	73 ± 4
*P*_gradient_ (mmHg)	2 ± 2	7 ± 3	6 ± 3
Effective orifice area (cm^2^)	1.1 ± 0.2	0.7 ± 0.2	0.8 ± 0.2

Data presented as mean ± standard deviation (SD)

*P*_*diastolic*_, diastolic pressure; *P*_*systolic*_, systolic pressure: from minimum and maximum aortic pressures; *P*_*gradient*_, transvalvular pressure gradient from peak aortic flow; *Q*_*mean*_, mean aortic flow

### Echography

Echographic measurements from the experiments are summarized in Table 2. In the long-axis view, there was a significant reduction in annular diameter during systole and diastole for both interventions when compared with the native group (Dacron ring group, *p* = 0.003; A-ring group, *p* = 0.020). There was no significant difference between the A-ring group and Dacron ring group in terms of reducing annular diameters during systole and diastole. The annular diameter in diastole was 14.7 ± 2.8 mm in the Dacron ring group and 15.8 ± 1.6 mm in the A-ring group. There was no significant difference in annular distensibility between all three groups. The relative annular distensibility was 9% in the native group, 13% in the Dacron ring group, and 10% in the A-ring group.

**Table 2:** Echographic measurements

	Native (*n* = 6)	Dacron (*n* = 6)	A-ring (*n* = 6)	ANOVA			*p*-values		
	Mean ± SD	Mean ± SD	Mean ± SD	DF	F	P	Native vs Dacron	Native vs A-ring	Dacron vs A-ring
Long-axis view									
Annulus diameter									
Systole (mm)	21.7 ± 2.3	16.9 ± 1.1	17.6 ± 1.5	2, 15	13.75	< 0.001	< 0.001	0.003	1.000
Diastole (mm)	19.8 ± 2.0	14.7 ± 2.8	15.8 ± 1.6	2, 15	9.06	0.003	0.003	0.020	1.000
Distensibility (mm)	1.9 ± 0.8	2.2 ± 2.2	1.8 ± 0.8	2, 15	0.19	0.831	ns*	ns*	ns*
Sinus diameter									
Systole (mm)	36.1 ± 2.7	30.6 ± 1.8	31.8 ± 2.7	2, 15	8.52	0.003	0.004	0.022	1.000
Diastole (mm)	34.9 ± 2.2	30.0 ± 1.8	30.6 ± 2.6	2, 15	8.54	0.003	0.005	0.015	1.000
Distensibility (mm)	1.2 ± 0.5	0.6 ± 0.3	1.2 ± 0.4	2, 15	3.82	0.046	0.079	1.000	0.105
Short-axis view									
Sinus area systole (cm^2^)	10.0 ± 1.6	6.4 ± 1.0	7.8 ± 1.8	2, 15	8.65	0.003	0.003	0.071	0.385
Sinus area diastole (cm^2^)	9.3 ± 1.4	6.3 ± 0.7	7.1 ± 1.6	2, 15	8.78	0.003	0.003	0.029	0.884
Sinus area distensibility (cm^2^)	0.7 ± 0.4	0.1 ± 0.3	0.7 ± 0.3	2, 15	6.54	0.009	0.017	1.000	0.025

Data presented as mean ± standard deviation (SD)

*DF*, degrees of freedom; *F*, *F*-statistics; *p*, *p*-value; ns*, non-significant ANOVA

At the level of the sinuses of Valsalva, there was also a significant reduction in systolic and diastolic diameters in both intervention groups, when compared with the native group. The diameters in the native group were 36.1 ± 2.7 mm in systole and 34.9 ± 2.2 mm in diastole. In the Dacron ring group, the sinus diameter was reduced to 30.6 ± 1.8 mm in systole and 30.0 ± 1.8 mm in diastole. Similarly in the A-ring group, the sinus diameter was reduced to 31.8 ± 2.7 mm in systole and 30.6 ± 2.6 mm in diastole. There was no significant difference between the A-ring group and the Dacron ring group at the sinus level in the long-axis view in both systolic and diastolic dimensions. There was no significant difference in sinus distensibility between all three groups.

**Fig. 4:**
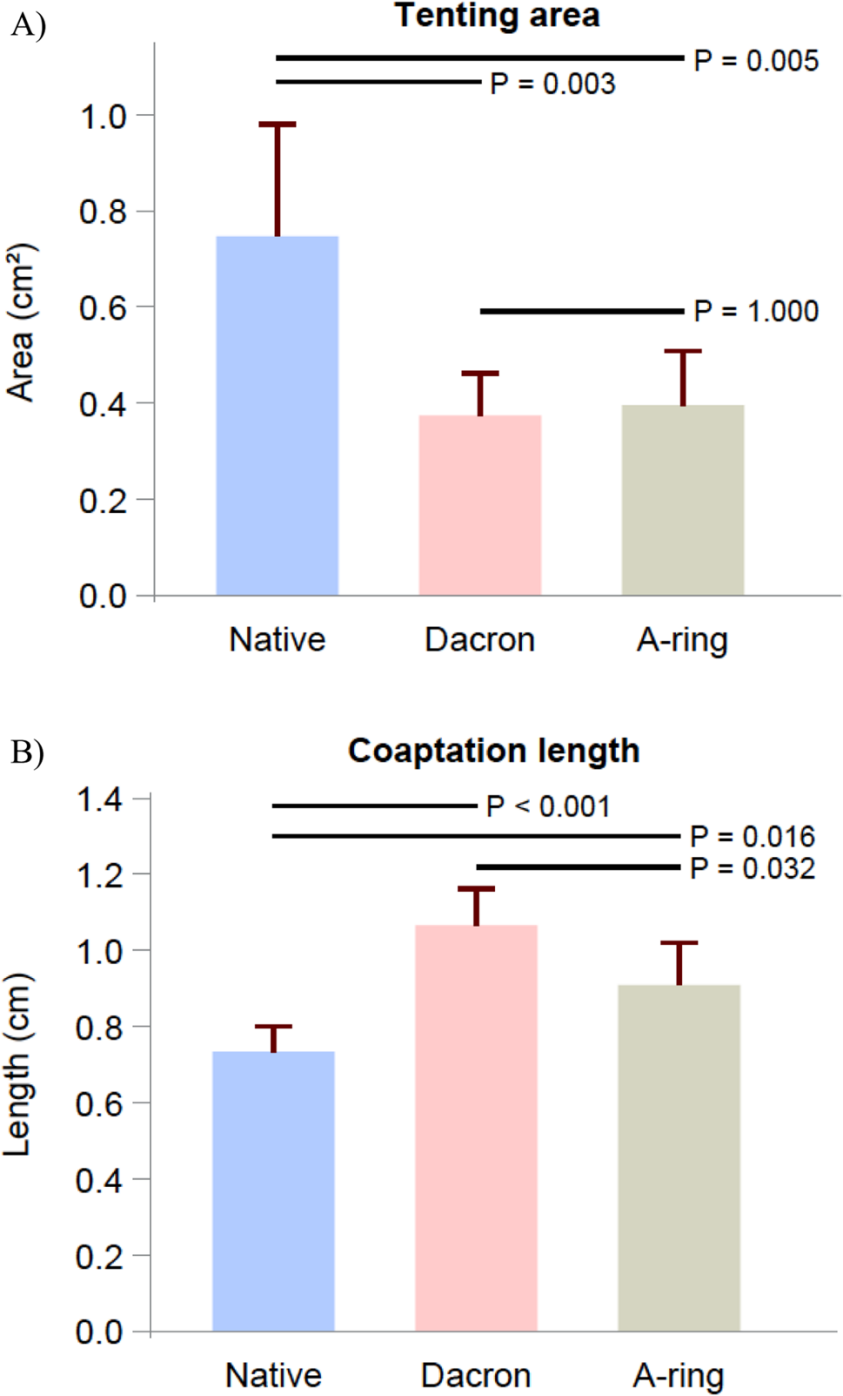

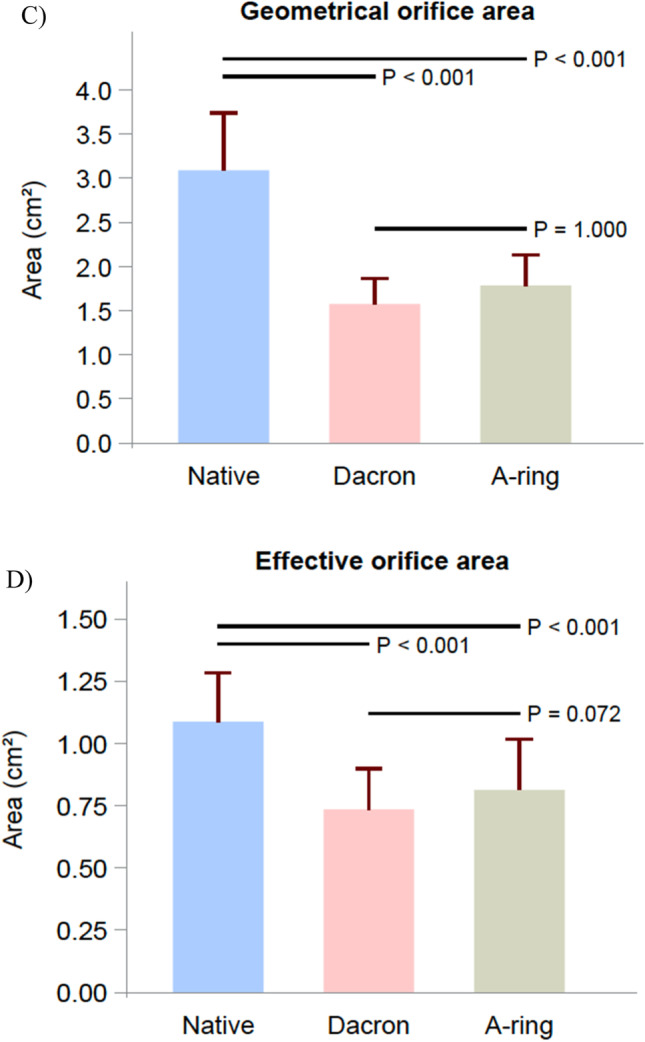
Echographic results and effective orifice area expressed as mean with standard deviation in the native, Dacron, and A-ring groups. **A** Tenting area. **B** Coaptation length. **C** Geometrical orifice area. **D** Effective orifice area

Coaptation length increased significantly in the Dacron ring group compared with the native group (*p* < 0.001). The A-ring group also significantly increased the coaptation length when compared with the Native group (*p* = 0.016). The Dacron ring increased the coaptation length significantly more than the A-ring (*p* = 0.032), as illustrated in Fig. 4.

Tenting area decreased in both the Dacron ring group and the A-ring group compared with the native group. The decrease was statistically significant for both intervention groups (Dacron group, *p* = 0.003; A-ring group, *p* = 0.005) when compared with the native group. There was no significant difference in tenting area between the A-ring group and Dacron ring group.

In the short-axis view, there was a reduction in sinus area for both interventions compared with the native group, however only statistically significant for the Dacron ring group during systole (*p* = 0.003). No significant difference was observed between the A-ring group and the native group in sinus area during systole (*p* = 0.071). Furthermore, there was no significant difference in sinus area during systole between the A-ring group and the Dacron ring group.

The sinus area distensibility was similar in the A-ring group and native group, whereas the Dacron ring exhibited a significantly lower distensibility compared with the native group (*p* = 0.017) and A-ring group (*p* = 0.025).

The geometrical orifice area decreased significantly in both intervention groups when compared with the native group (*p* < 0.001). No significant difference in geometrical orifice area was observed between the A-ring and the Dacron ring group.

## Discussion

In this study, we evaluated a new open aortic annuloplasty ring, the A-ring, and compared it with the Dacron ring. The comparison was based on aortic root dimensions and dynamics by two-dimensional echography along with hydrodynamic assessment. The study demonstrates the effect of a Dacron ring and novel A-ring on aortic root dynamics compared with the native aortic root.

Overall, the echographic data revealed that both interventions had a downsizing effect on the aortic root, while still maintaining the distensibility comparable to the native condition. The results were consistent with findings from other similar in vitro and in vivo studies investigating aortic annuloplasty procedures [7, 23].

There was no statistically significant difference in annular distensibility between any of the groups ranging from 9 to 13%, which is within the normal range of distensibility of the native aortic root [8].

Both interventions were intended to downsize the aortic annulus and thus, an increase in the transvalvular gradient was seen after both interventions. Although the measured values of the pressure gradient did not reach clinically relevant values, it was still possible to detect a statistically significant difference between the groups due to the inherent very low beat-to-beat variation in vitro.

In a clinical evaluation of advanced surgery, such as aortic valve repair, it is challenging to identify and evaluate the independent effect of an annuloplasty ring due to multiple coexisting influencing factors, such as anatomy, comorbidities, and pathology [17]. Moreover, surgical skills vary among surgeons, which potentially may also lead to different clinical outcomes. These confounding factors are in this in vitro study limited by having the same surgeon for all procedures. This study is the first to relate and compare the independent functional outcome of the novel A-ring and the Dacron ring compared with the native aortic root under controlled reproducible conditions in an in vitro model.

As part of the recommendations for treating valvular heart disease in young patients with aortic root dilatation, the European Society of Cardiology and the European Association of Cardiothoracic Surgery have included external aortic annuloplasty alongside the remodeling procedure [4, 24]. Studies suggest that aortic valve repair should resemble the physiological behaviors seen in healthy aortic roots [4, 16]. Thus, a target for an in vivo expansion of the A-ring was made to be between 5 and 15% strain. Since our proposed novel A-ring has an in vitro distensibility within this range, it could be considered to be an applicable adjunct used in aortic valve-sparing procedures. Furthermore, it has the advantage of being an open annuloplasty ring, which will alleviate surgeons from detaching and reimplanting the coronary arteries for isolated aortic valve repair.

It was hypothesized that the A-ring would downsize and stabilize the aortic root equally to the Dacron ring. This study supports the hypothesis and further shows that the A-ring has comparable supportive characteristics like the Dacron ring despite having different material properties. A more detailed investigation using the same A-ring in vivo is currently ongoing to further evaluate hemodynamic performance and consistency. However, the current study suggests that the novel A-ring has the potential capabilities to be used as a relevant annuloplasty ring for aortic valve repair based on its acute behavior and functionality evaluated in vitro.

### Limitations

The in vitro model has the ability to replicate physiological conditions under controlled and reproducible conditions. Nonetheless, an in vitro flow loop has certain limitations concerning the use of the aortic roots coupled to non-physiological material components. In order to attach the aortic roots in the flow loop, the left ventricle of the hearts was removed so that the aortic roots could be attached. This resulted in a rigid non-flexible left ventricular outflow tract that could affect our measurements. However, all roots were investigated under identical conditions which is a strength in terms of comparison. Porcine aortic roots and hearts are generally used and accepted for experimental studies in cardiac surgery. However, one drawback is that porcine hearts have certain anatomical differences compared with the human heart. Additionally, all the hearts were harvested from healthy pigs and accordingly had no prior aortic root pathology.

## Conclusion

The Dacron ring and A-ring both effectively downsized the aortic annulus in this in vitro study. Both annuloplasty procedures significantly increase coaptation length and caused a significantly reduction in tenting area. Furthermore, the novel A-ring revealed similar aortic root dynamics to the Dacron ring with the ability to maintain aortic root distensibility and hemodynamic performance during the cardiac cycle.

### Supplementary Information

Below is the link to the electronic supplementary material.

Supplementary file1 (DOCX 978 KB)

Supplementary file2 (DOCX 61 KB)

## Abbreviations

EOA: Effective orifice area
GOA: Geometric orifice area
