# Supplementary material for: Novel Expansible Aortic Annuloplasty Ring Exhibits Similar Characteristics as the Dacron Ring—an In Vitro Evaluation
Source: J Cardiovasc Transl Res. 2023 Jun 1;16(5):1144–52. doi: 10.1007/s12265-023-10393-7 (PMC10615915; doi:10.1007/s12265-023-10393-7)
Supplement: Supplementary file 2 — Supplementary file2 (DOCX 61 KB) [file 12265_2023_10393_MOESM2_ESM.docx]

**Novel expansible aortic annuloplasty ring exhibits similar characteristics as the Dacron ring – an *in vitro* evaluation**

**Intra-and interobserver variability**

The method for calculating intra and interobserver variability in this study was based upon this paper:

Popović ZB, Thomas JD. Assessing observer variability: a user's guide. Cardiovasc Diagn Ther. 2017 Jun;7(3):317-324. doi: 10.21037/cdt.2017.03.12. PMID: 28567357; PMCID: PMC5440257.

Observers:

- For the study there were 2 observers: Observer 1 and Observer 2.
- Each of the observers performed the echocardiographic measurements on all 18 aortic roots independently and blinded.
- Observer 1 performed the echocardiographic measurements on two different occasions. And the intraobserver variability was based upon the two measurements.
- Assessment of interobserver variability: samples. The first measurement of observer 1 was paired with the measurements made by Observer 2.
- Both absolute and relative intra- and inter-observer variability was calculated using the method described in the paper:

Because there were only 1 set sample of images this method was deemed to be the right one.

Table 1.


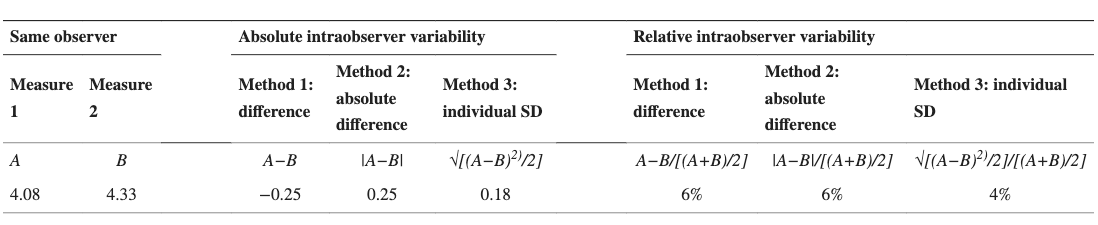


Method:

- All echographic parameters were calculated in this manner
- Method 1 contains individual differences, and calculating the mean and standard deviation of those differences.
- Method 2 calculates the absolute value of individual differences, and calculating the mean and standard deviation.
- Method 3 involves calculating the standard deviation of individual pairs of measurements, and then calculating the mean and standard deviation of the third column.
- Reporting in actual measurement units or percentages depends on the characteristics of the measurement error.
- Both methods have been included in the report.
